# Do people with HIV really have the choice between oral and injectable ART? Evidence from a multicentric survey in the Swiss HIV cohort study

**DOI:** 10.1111/hiv.70239

**Published:** 2026-04-08

**Authors:** Yonas Martin, Bernard Surial, Martin Müller, Lukas Baumann, David Jackson‐Perry, David Haerry, Marie Ballif, Charles Béguelin, Miriam Bürgi, Aline Munting, Matthias Cavassini, Dominique Braun, Andri Rauch, Reto Auer, Gilles Wandeler, the Swiss HIV Cohort Study

**Affiliations:** ^1^ Department of Infectious Diseases Inselspital, Bern University Hospital, University of Bern Bern Switzerland; ^2^ Department of Emergency Medicine Inselspital, Bern University Hospital, Bern University Bern Switzerland; ^3^ Service of Infectious Diseases Lausanne University Hospital and University of Lausanne Lausanne Switzerland; ^4^ Positive Council Switzerland Zurich Switzerland; ^5^ Institute of Social and Preventive Medicine, University of Bern Bern Switzerland; ^6^ Department of Internal Medicine Spitalzentrum Biel Biel/Bienne Switzerland; ^7^ Hirslanden Klinik Zurich Switzerland; ^8^ Institute of Primary Health Care (BIHAM), University of Bern Bern Switzerland; ^9^ Centre for Primary Care and Public Health (Unisanté) Lausanne Switzerland

**Keywords:** antiretroviral agents, HIV, injectable ART; shared decision making, long‐acting ART, patient preference

## Abstract

**Introduction:**

Injectable long‐acting antiretroviral therapy (iLA‐ART) offers a valuable alternative to oral ART (oART). While the efficacy of these treatment strategies is similar, adequate information on their specific characteristics is essential to enable people with HIV (PWH) to decide which option best suits their values and preferences.

**Methods:**

We conducted a multicentric survey of PWH on oART in the Swiss HIV Cohort Study (SHCS). Using a questionnaire co‐developed with expert patients, we assessed participants' (1) values and preferences on characteristics of modern oART and treatment satisfaction, (2) knowledge about iLA‐ART with cabotegravir/rilpivirine, (3) reasons influencing their interest to switch or not to iLA‐ART and (4) perceived burden of treatment (BOT) taking oART. Outcomes were rated on an 11‐point (0–10) Likert scale. We explored outcomes' determinants using multivariate analyses.

**Results:**

A total of 200 PWH on oART participated (response rate 87%), with a median age of 52 years (Interquartile Range 45–59), 58 (29%) were women, and 90 (45%) were men who have sex with men. Treatment satisfaction was very high (mean 9.3, Standard Deviation [SD] 1.3) and perceived BOT on oART was low (mean 2.5, SD 2.0). The two most valued oART characteristics were effectiveness (mean 9.9, SD 0.3) and absence of side effects (mean 9.5, SD 1.7). Overall, 76 (39%) participants had never heard about iLA‐ART, with large differences between the 3 participating centres (60% vs. 3% vs. 50%). In multivariable analysis, women (Odds Ratio 0.35, 95% confidence interval [CI] 0.14–0.85) and PWH ≥60 years (0.27, 0.08–0.94) were less aware about iLA‐ART. Reasons influencing PWH's potential interest to switch to iLA‐ART varied individually, while the main reason for preferring to stay on oART was the bimonthly dosing interval of iLA‐ART.

**Conclusion:**

In Switzerland, over one‐third of PWH were unaware of iLA‐ART despite its reimbursed availability. The provider of care appears to be the main driver of these findings, while women and older individuals showed the lowest awareness. As ART characteristics are valued individually, providing systematic information on available treatment options and engaging PWH in shared decision‐making could help address identified disparities and empower them to choose the treatment that best aligns with their preferences.

## INTRODUCTION

Injectable long‐acting antiretroviral therapy (iLA‐ART) with cabotegravir (CAB) and rilpivirine (RPV) has transformed the treatment for HIV in recent years and this treatment option is available and reimbursed since March 2022 in Switzerland [1, 2, 3, 4]. iLA‐ART represents an attractive alternative to oral ART (oART) for several reasons. Indeed, adherence to oART can be challenging for some individuals, for instance due to pill count or size, and disclosure or privacy concerns associated with HIV‐related stigma [5]. Qualitative studies have shown that when asked about iLA‐ART, people with HIV (PWH) reported improved convenience, freedom, greater confidentiality and the psychosocial and emotional benefits associated with not being constantly reminded of living with HIV [6, 7]. Clinical trials have shown high acceptability of iLA‐ART with CAB/RPV, but study participants may have had a high a priori interest in iLA‐ART [6, 8]. Indeed, a survey among 374 PWH conducted in the United States showed that 41% of participants preferred continuing oART rather than switching to iLA‐ART [9]. Another survey showed that the strongest driver influencing PWH's willingness to switch to iLA‐ART is a longer dosing interval: 42% would choose this option if administered every 6 months versus only 7% if administered bimonthly [6].

There are other reasons why PWH might not wish to switch to currently available iLA‐ARTs. Concerns were raised about the potential for stigma associated with clinic attendance for injections, for instance, if colleagues notice PWH missing work frequently for appointments [7]. Furthermore, some people might fear discomfort associated with intramuscular injections. Finally, PWH who have other chronic conditions and are taking additional medications may not see major reductions in their daily pill burden. ILA‐ART implementation is also challenged by time constraints, both at the patient and clinical level, that is in a busy HIV care setting [10]. The well described increased burden on health care staff associated with iL‐ART implementation has even been identified as a barrier for providers to offer this treatment option [11, 12].

The choice between oART and iLA‐ART depends on how individuals value the burden, risks and benefits of each treatment. Because treatment options are equally efficacious, this equipoise is described as preference‐sensitive [13]. In such situations, shared decision‐making (SDM) is ideal to help patients choose their preferred option [14]. Applying SDM increases PWH's treatment readiness and confidence, their understanding of the importance of adherence and enhances the provider‐patient relationship [15, 16, 17]. Also, substantial variations in medical care have been consistently reported, particularly in preference‐sensitive health decisions [18, 19, 20, 21]. Such variations are often not explained by objective factors and tend to reflect providers' practices rather than patients' preferences [13, 22, 23]. SDM also helps reduce unwarranted variation in care by decreasing variation between providers, while promoting appropriate variation within them.

To support treatment choices among PWH, a good understanding of their preferences and literacy about current ART strategies is warranted. We thus explored treatment satisfaction, preferences, values about oART characteristics, knowledge about iLA‐ART, reasons influencing for or against a potential switch to iLA‐ART and burden of treatment among PWH on oART in the Swiss HIV Cohort Study (SHCS).

## METHODS

### Study setting and population

We conducted a multicentric paper‐based survey at three HIV clinics associated with the SHCS between November 2022 and January 2024. The different centre settings (size, language region, university vs. non‐university) were chosen to ensure representativeness of participants. Centres 1 and 2 are large university hospitals situated in the German‐ and French‐speaking regions of Switzerland, respectively. Centre 3 is a medium‐sized non‐university hospital located in a bilingual German/French‐speaking region. The SHCS is a nationally representative prospective cohort study and includes approximately 80% of all PWH receiving ART in Switzerland [24]. Demographic, clinical and laboratory data as well as changes in ART regimens, comedications and behavioural data are prospectively recorded at registration and every 6 months thereafter, using a standardized protocol. All centres' local ethical committees approved the cohort study, and all patients provided written informed consent. This specific study fell within the scope of the general ethical approval of the SHCS.

We included PWH aged ≥18 years on oART and excluded those with contraindications for iLA‐ART (Supporting Information File S1) because we felt it was unethical to engage in discussions on iLA‐ART with PWH who wouldn't have the option to switch to this strategy. To reduce selection bias, centres were instructed to enrol participants at random using locally determined procedures to ensure feasibility. If PWH accepted to participate, the questionnaire was filled out by the participant, with the support of study nurses if needed. Participants needed to be fluent enough in French, German or English as the questionnaire was available in these three languages.

### Outcome measures and data collection

The survey entailed the following outcomes sections: (1) values and preferences about characteristics of modern oART and treatment satisfaction, (2) knowledge about iLA‐ART with CAB/RPV, (3) reasons for or against a potential switch to iLA‐ART and (4) burden of treatment (BOT) associated with oART.

We co‐produced the survey tool in a team consisting of clinician‐researchers and expert patients (patient and public involvement [PPI] members) and pilot‐tested the questionnaire among three PWH representatives. Answers to Sections INTRODUCTION, RESULTS and 4 were collected using an 11‐point (0–10) Likert scale enabling discrimination of nuances and making a neutral answer (answer ‘5’) possible. In addition, the possibility to having ‘no opinion’ was given. Section 2 was a multiple‐choice section covering awareness about iLA‐ART and 10 specific knowledge questions. The full version of the questionnaire is accessible in (Supporting Information File S2).

Participants' characteristics were extracted from the SHCS data set, which simplified questionnaire use and implementation.

### Study evaluations and data analysis

We assessed the representativeness of our study population by comparing sociodemographic and virological characteristics with the overall eligible population across the three SHCS centres. Differences between these samples were tested using Chi‐square tests for categorical variables and student *t*‐test for continuous variables.

**FIGURE 1:**
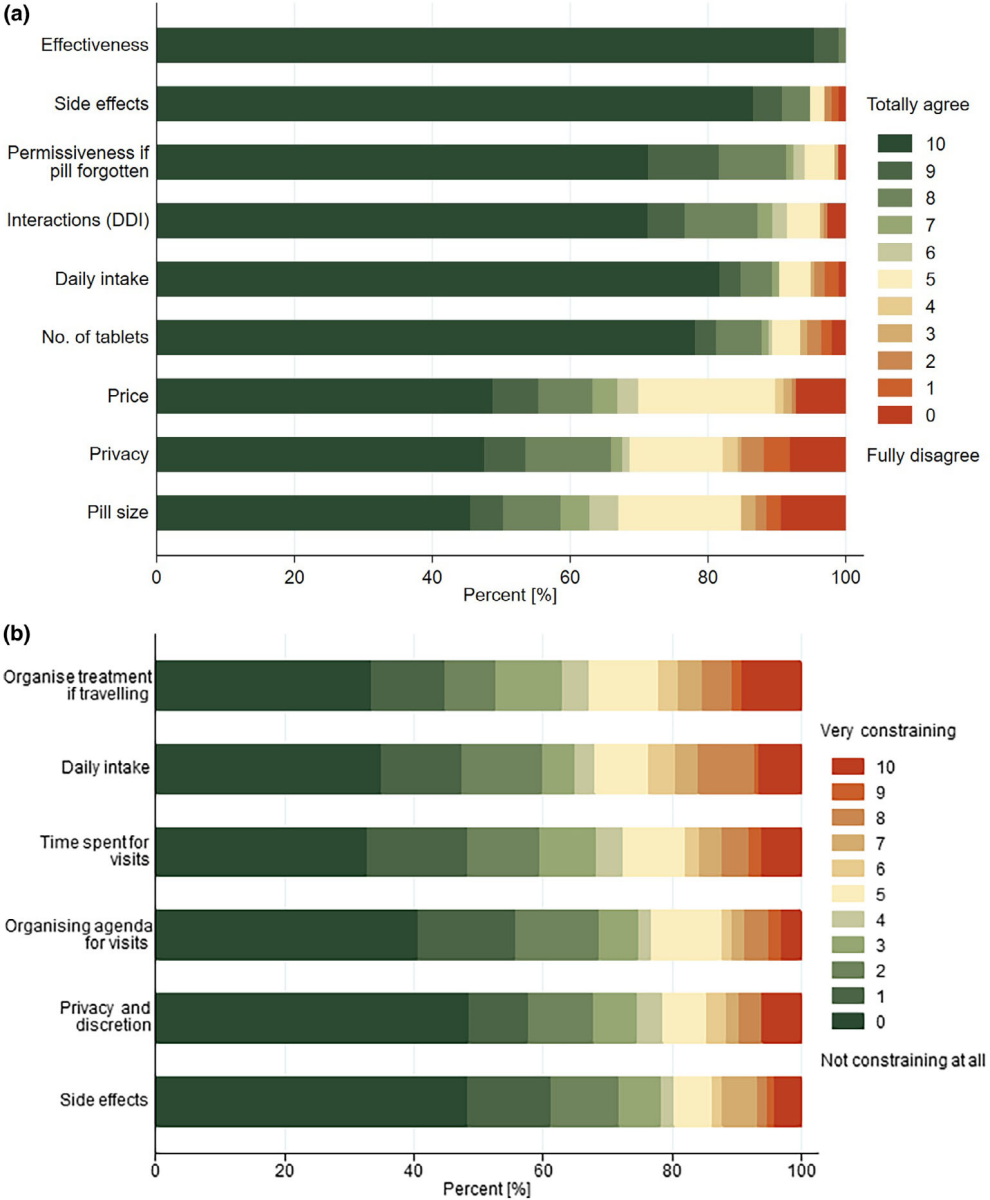
Panels A and B. Panel A. Reported importance of oral ART characteristics, *n* = 200. Stacked bar chart showing the distribution of answers (Likert scale 0–10) of participants for each ART characteristic item. The colour code assigned to each possible answer facilitates visual representation of this distribution. Items are sorted according to their mean score, the first being overall valued as the most important characteristic and the last item at the bottom valued as the least important one. DDI: Drug‐drug interactions. Panel B. Reported burden of treatment (BOT) associated with oral ART characteristics, *n* = 200. Stacked bar chart showing the distribution of answers (Likert scale 0–10) of participants for each BOT item. The colour code assigned to each possible answer facilitates visual representation of this distribution. Items are sorted according to their mean score, the first being overall valued as the most constraining characteristic and the last item at the bottom valued as the least constraining one.

**FIGURE 2:**
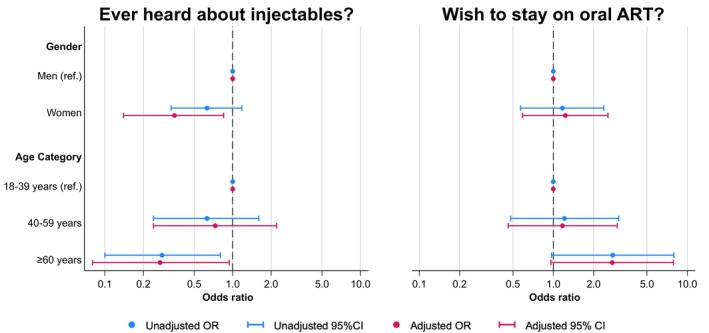
Uni‐ and multivariable analyses of awareness about iLA‐ART and future treatment intentions. Forrest plots showing uni‐ and multivariable analyses of (1) ‘having previously heard about injectables’ (multivariable analyses adjusted for gender, age, level of education and centre) and (2) **‘**future treatment intentions’ (multivariable analyses adjusted for gender and age). In multivariable analyses, women and participants aged ≥60 years were less likely to have heard about iLA‐ART before. Participants ≥60 years tended to be more likely to intend to stay on oART, although this trend was statistically non‐significant.

**FIGURE 3:**
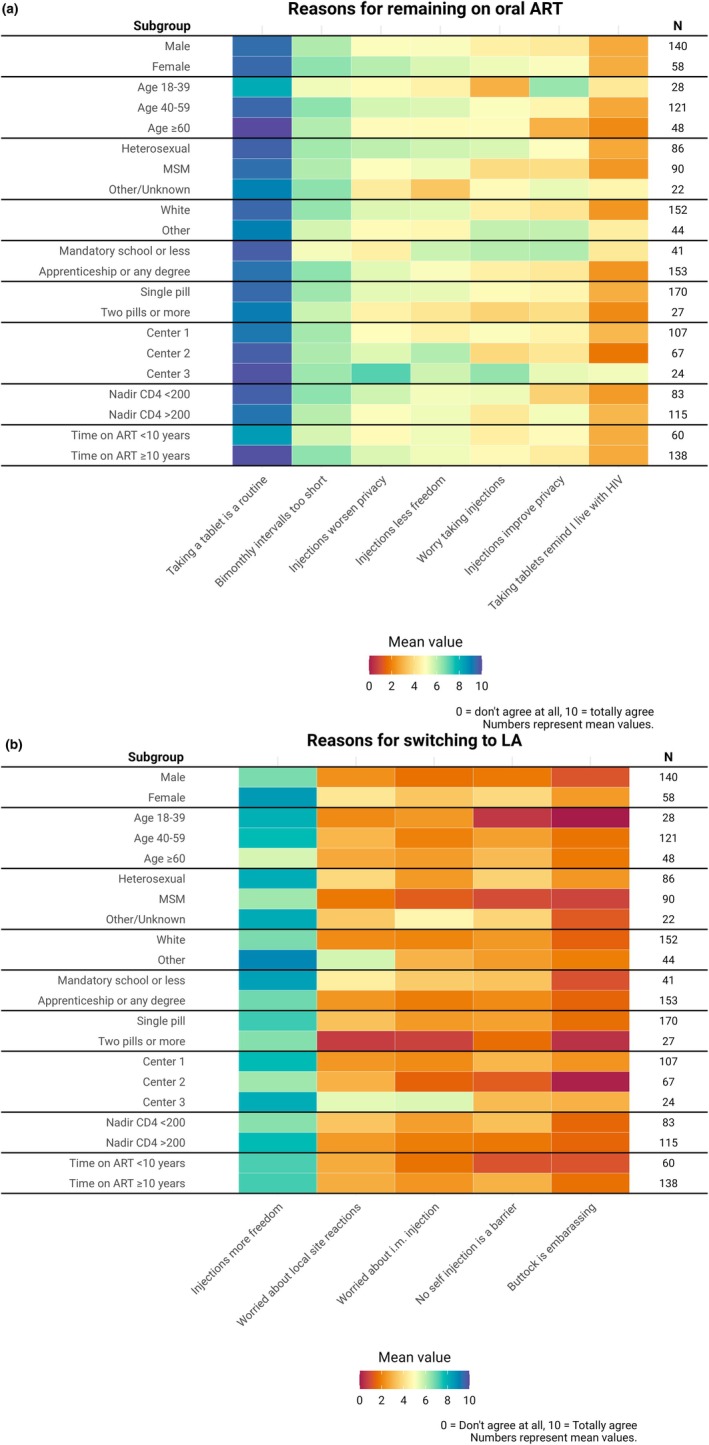
Panel A and B. Panel A and B. Heatmaps of reasons for remaining on oral ART (**Panel A**) or reasons for being interested in a potential switch to iLA‐ART (**Panel B**) across participant subgroups (*y* axis). Total participants (*N* = 200) rated their agreement/disagreement with different statements (*x* axis) on a Likert scale from **0** (‘**don't agree at all**’) to **10** (‘**totally agree**’). For space reasons, a short version of the statements (y axis) was chosen to be included in the Figure, the full version is available in the questionnaire (Supporting Information File 2). Colours represent mean scores for each subgroup, with each colour assigned to one value (blue/purple represents the strongest agreement and red the lowest). Numbers on the right indicate subgroup sample sizes. This representation offers granular and descriptive visual information, allowing comparison between subgroups of interest while considering their sizes. Due to the small subgroup sizes and the exploratory nature of the outcomes we decided to present these results without a statistical test. **Example of interpretation of Panel A:** As an example, there is a consistently strong agreement across subgroups that ‘taking a pill is a routine’, whereas younger participants (18–39 y.o.) seem to value this statement in a more nuanced manner. However, this subgroup appears less ‘worried to take injections’ than the others. **Example of interpretation of Panel B:** Participants on oral ART with 2 pills or more seem to be less worried about potential LA‐ART‐associated reactions and about intramuscular injections than participants on single‐pill oral ART.

Categorical variables were expressed as frequencies, and continuous variables were expressed as means and standard deviations (SD) or medians and interquartile ranges (IQR). Using logistic or linear regression, we tested the univariable association between the outcomes and the participants' sociodemographic characteristics (gender, age, transmission group, ethnicity, highest level of education, centre of care) or virologic factors (current ART, CD4 nadir, ART duration). Gender and age, as well as other factors associated with the outcomes in univariable analyses with a p value <0.1 were included in the multivariable models. Statistical analyses, Figures 1 and 2 were done using STATA 16.0 (StataCorp, College Station, TX) and Figure 3 was produced in R, version 4.2.1 (R Foundation for Statistical Computing).

### Patient and public involvement (PPI)

The questionnaire was developed in collaboration with healthcare providers and PWH, as patients can best determine which health outcomes are relevant for them [25, 26, 27, 28, 29, 30, 31] and whether questionnaires capture these outcomes in a comprehensible and understandable manner [31, 32, 33]. Our research team included two expert PWH, both scientific board members of the SHCS. They were consulted before defining research questions and were involved in the conception of the research proposal as well as the questionnaire and writing this manuscript. They were also involved in discussions regarding the budget allocated for PPI and were informed about relevant aspects during the study completion. Three additional members of the HIV Community Council at the University Hospital of Lausanne (Switzerland) edited and tested the questionnaire. Their diverse sociodemographic backgrounds helped to improve readability, facilitate comprehension and increase representativeness. According to guidance in this journal, GRIPP2 short form has been used for detailed PPI reporting (Supporting Information File S3) [34].

## RESULTS

Of the 231 eligible PWH asked to participate, 200 (87%) accepted to fill out the questionnaire. The acceptance rate was 83% (107/129) in Centre 1, 88% (67/76) in Centre 2, and 100% (26/26) in Centre 3. Median age was 52 years (IQR 45–59), 58 (29%) participants were women, 90 (45%) were men who have sex with men (MSM) and 153 (77%) were white (Table 1). Our study sample was representative of the eligible population across the 3 centres for most of patients' sociodemographic characteristics, but included slightly more white PWH (77% vs. 69%), PWH with higher education (78% vs. 70%), PWH on single pill regimen (86% vs. 70%), PWH from Centre 1 (53% vs. 41%) and PWH with suppressed viremia (98% vs. 94%) than in the eligible population (Supporting Information File S4).

**TABLE 1:** Patients' sociodemographic and virologic characteristics.

Characteristic	Total *n* = 200 (%)
Women	58 (29)
Age category	
18–39 years	28 (14)
40–59 years	122 (61)
≥60 years	50 (25)
Transmission group	
Heterosexual	88 (44)
PWID	12 (6)
MSM	90 (45)
Other	8 (4)
Missing	2 (1)
Ethnicity	
White	153 (77)
Other	45 (23)
Missing	2 (1)
Highest level of education	
Mandatory school or less	42 (22)
Apprenticeship or any degree	153 (78)
Missing	5 (2)
Current oART	
Single tablet regimen	172 (86)
At least two pills	27 (14)
Missing	1 (1)
Centre	
1	107 (53)
2	67 (34)
3	26 (13)
Last HIV viral load	
<50 cp/mL	195 (98)
>50 cp/mL	5 (2)
CD4 nadir	
<200 cells/μL	85 (43)
≥200 cells/μL	115 (57)
ART duration, median (IQR)	14.3 (8.6–20.0)
<10 years	61 (31)
≥10 years	139 (69)

Abbreviations: ART, antiretroviral therapy; MSM, Men who have sex with men; PWID, People who inject drugs.

### Values and preferences about oART characteristics, burden of treatment and treatment satisfaction

Values and preferences about oART characteristics are represented in Figure 1 *—Panel A*. The two most valued ART characteristics were: Effectiveness (mean 9.9, SD 0.3) and side effects (‘as few as possible’, mean 9.5, SD 1.7). The two least valued ART characteristics were pill size (‘as small as possible’, mean 7.2, SD 3.3) and ensuring privacy (flexible intake time, size of the package, mean 7.3, SD 3.4).

The overall perceived BOT of oART was low (mean 2.5, SD 2.0), even among patients who reported to be interested in knowing more about iLA‐ART (mean 2.7, SD 1.9). The answers to the BOT questions are represented in Figure 1 *—Panel B*. The item valued as being associated with the highest BOT was organizing treatment in case of travelling, prolonged leave or missed appointment (mean 3.2, SD 3.3), whereas side effects were valued as the least burdensome (mean 2.0, SD 2.9). In multivariable analyses, a higher level of education was associated with lower overall BOT (participants with apprenticeship or any degree vs. mandatory school or less: Coeff. −1.0; CI −1.8 to −0.3). Similarly, participants from Centre 2 reported a lower overall BOT compared to participants from other Centres (Coeff, −0.9; CI −1.6 to −0.3; Ref: Centre 1).

Full multivariable analyses on BOT are shown in Supporting Information File S5.

Mean satisfaction with current oral treatment was high (9.3, SD 1.3). In multivariable analyses (covariates: gender, age, CD4 nadir), being aged ≥40 years (reference age 18–39 years) was associated with higher mean treatment satisfaction (40–59 years: Coeff 0.9, CI 0.3–1.4; ≥60 years: Coeff. 0.9, CI 0.3–1.5).

### Patients' knowledge about iLA‐ART

Overall, 76 (39%) participants reported having never heard about iLA‐ART, with large differences between centres (Centre 1: 60%, Centre 2: 3%, Centre 3: 50%). In multivariable analyses, being a women (OR 0.35, CI 0.14–0.85) or aged ≥60 years (OR 0.27, CI 0.08–0.94, *p* = 0.052) were associated with lack of awareness about iLA‐ART (Figure 2). Participants treated in Centre 2 were more likely to have heard about iLA‐ART previously compared with the other centres (OR 52.0, CI 11.5–235.7 for Centre 2 vs. Centre 1). Among participants who stated having previously heard about iLA‐ART (*n* = 118), only 47% knew that the effectiveness of iLA‐ART was comparable to modern oART, 61% knew that iLA‐CAB/RPV had to be injected bimonthly (vs other intervals), 39% knew that the drugs had to be given intramuscularly and 53% knew that iLA‐CAB/RPV had to be injected in the buttocks. Of all participants who mentioned having heard about iLA‐ART and who answered all 10 specific knowledge questions (*n* = 111), only 59 (53%) answered correctly to ≥50% of the questions. Full multivariable analyses for previously ‘*having heard about injectables*’ are shown in Supporting Information File S6.

### Reasons for or against a potential switch to iLA‐ART

Overall, 63 (31**%**) participants mentioned they would prefer to stay on oART, whereas 98 (49**%**) would like to know more about iLA‐ART, and 33 (17%) said they did not know. Multivariable analyses on ‘Factors associated with willing to stay on oral ART’ did not show any differences across subgroups (Supporting Information File S7). The most commonly mentioned reasons for participants wishing to stay on oART were (1) that taking a pill is a routine (mean 8.8, SD 2.4), and (2) that bimonthly visits would not be desirable (mean 6.0, SD 3.7). Interestingly, fear of intramuscular injections (mean 3.1, SD 3.6) or local site reactions (mean 3.6, SD 3.6) and perceiving the buttocks as an embarrassing site of injection (mean 2.7, SD 3.3) seemed to be minor concerns. Figure 3 Panel A/B presents a heat map illustrating how subgroups of participants valued specific characteristics of oART or iLA‐ART.

## DISCUSSION

Our study shows that in Switzerland, treatment‐related satisfaction of PWH on oART is high and that some oART characteristics such as effectiveness or absence of side effects are valued as of utmost importance. Strikingly, despite being available and reimbursed for more than 6 months before the survey began, over one‐third of PWH were unaware of the existence of iLA‐ART, and women and older PWH appeared to be particularly affected by this knowledge gap. As wide centre differences were identified, the way treating physicians counsel PWH on available treatment options seems to be the main driver of these results. Ultimately, the reasons for or against a potential switch to iLA‐ART were valued highly individually, underscoring the need for a tailored approach towards SDM discussions that comprehensively present the current therapeutic strategies.

Our results showing high overall satisfaction with oART are in line with a previous Spanish study among 602 PWH, which found a mean satisfaction level of >8.5 on a 10‐point scale [35]. Efficacy and a favourable side effects profile seem to be universally perceived as being of major importance as shown by several studies conducted on different continents in various settings [35, 36, 37, 38, 39]. We could hypothesize that the high level of satisfaction found is at least partly explained by these two driving characteristics, as modern oART meets them in most of the cases. While PWH are understandably not keen to make any compromise on these characteristics, other features such as pill size and privacy seem to be generally of lower importance; however, with more interindividual differences. These results highlight the importance of individualized therapeutic approaches rather than ‘one‐pill fits all’ regimes for PWH.

We found that awareness about iLA‐ART was generally low and varied across subgroups, particularly across gender and age. Gender and sex inequity in healthcare has been increasingly recognized and studied over the past decades. Several groups have investigated the determinants and the reach of gender inequities, not only showing under‐representation of women in various fields of clinical research [40, 41, 42, 43] but also pointing out discrepancies in terms of access to care and population outcomes [44, 45, 46, 47, 48]. More specifically in the field of HIV, a systematic review described well the major under‐representation of women in trials on antiretrovirals and cure strategies [49]. Similar evidence has been found in terms of age, with older individuals being at risk for inequities in healthcare and research [50, 51, 52, 53, 54, 55]. Our study is the first describing gender and age inequity in terms of awareness about iLA‐ART. The observed gender gap is of particular concern as women do not appear to show less interest in iLA‐ART than men, according to our results and other studies [56, 57]. These results should increase awareness among persons involved in HIV care, and every effort should be made to close such inequities. We hypothesize that the lower level of awareness observed among women and older individuals may be a direct consequence of unequal provision of information by HIV physicians, potentially reflecting centre‐specific practices. Obvious structural differences (programmatic vs. opportunistic counselling on iLA‐ART) could be identified by our team and are likely to open the door to such variation in care. In an opportunistic setting, implicit bias might be one relevant determinant of inequities and different strategies have been suggested to address this bias, such as educational and informative approaches targeting health care personnel [52, 58, 59, 60]. In light of these results, centres using opportunistic counselling should consider strategies to ensure that all PWH get the chance to be informed about available treatment options. In addition, given that peer support and online HIV resources have been shown to positively influence several key aspects of the lives of PWH, including HIV‐related knowledge, these options should be routinely considered [61, 62, 63, 64].

In line with the available literature, we found that some therapy‐related characteristics seem to be important drivers for people's decision to decline iLA‐ART [6, 65, 66, 67, 68]. A ‘short’ dosing interval (bimonthly) or switching away from a therapy strategy that is well integrated in the daily routine (oART) are illustrative examples of such characteristics. The proportion of PWH potentially interested in switching to iLA‐ART in Switzerland is comparable to previous findings in other countries, but is much lower than in the first report dating from 2013, in which 84% of 400 surveyed PWH in the United States mentioned they would ‘definetely’ or ‘probably’ try iLA‐ART if a monthly dosing interval was available [69]. This difference highlights the evolving character of patients' values and preferences over time, while major improvements in oART have been synchronously achieved. When exploring how subgroups value specific reasons for or against a potential switch to iLA‐ART, certain trends appear to emerge. For example, the perceived impact on privacy of a potential switch to iLA‐ART seems to differ across age groups. Also, apprehension about taking injections (‘worry taking injections’) appears to be differently weighted between men and women, MSM and heterosexual, younger PWH versus older ones and PWH with vs. without higher education. Finally, fear of potential local site reactions seems to vary between white participants versus participants of other ethnicities. However, in view of the small subgroups sizes and explorative nature of these results, the potential differences must be interpreted with caution. These findings might be suggestive of how each participant individually balances iLA‐ART characteristics and highlight the importance to engage in SDM discussions with PWH, helping to identify their values and preferences in terms of therapy strategy.

To our knowledge, our study is among the first to adress awareness and knowledge about injectable LA‐ART. Further strenghts of our study include the fact that it is patient‐centred in its essence. The study included a strong multistage PPI component, allowing the co‐production of the survey questionnaire with subsequent improvement by a team including expert patients and members of a comunity council with different sociodemographic backgrounds. Also, we decided to use a 10‐point Likert scale allowing discrimination of nuances and the possibility of giving a neutral answer. Although we sought to include participants at random, the centre's freedom to decide how they recruited participants may have led to some selection biais within and across centres. However, except for some characteristics and to a minor extent only, the study sample was representative of the eligible population at the three centres. Also, the small subgroup sizes and explorative nature of our results on the perceived reasons for or against a potential switch to iLA‐ART don't allow us to draw any conclusions on the potential subgroup differences. Finally, some specific groups—such as pregnant or transgender people with potentially particular concerns (e.g., iLA‐CAB/RPV not approved in pregnancy; potential impact of gluteal implants or fillers on iLA‐ART absorbtion)—were not represented, and therefore no conclusion can be drawn for these populations.

## CONCLUSIONS

In Switzerland, satisfaction with oART is high and while some ART characteristics are reported being of utmost importance, others are valued with more nuance. Our study identifies significant information gaps on iLA‐ART, in particular women and older PWH being less aware about this treatment option. Finally, while a few reasons seem to drive decisions for switching to iLA‐ART, many reasons are weighted more individually. These results should help clinicians address inequity of PWH's awareness and knowledge on available treatment options giving them the chance to make informed decisions. Further, our findings help to better understand how and why PWH decide for or against different treatment strategies. This, in turn, helps lay the groundwork for developing decision aids that support physicians in engaging PWH in SDM discussions about ART options. Such tools ultimately enable individuals to choose the treatment strategy that better matches their preferences.

## AUTHOR CONTRIBUTIONS

Y.M., B.S., A.H., D.JP., D.H., M.B., C.B., Mi.B., A.M. and M.C. performed the research. Y.M., B.S., D.JP., D.H., M.C., A.R., R.A. and G.W. designed the research study. Y.M., B.S., D.JP., D.H., and G.W. contributed essential tools (questionnaires). Y.M., B.S., M.M., L.B. and G.W. analysed the data. Y.M. wrote the paper.

## FUNDING INFORMATION

This work was performed within the framework of the Swiss HIV Cohort Study, which offered a project grant # 899 to support this work. The Swiss HIV Cohort Study is supported by the SNSF grant #201369, and by the SHCS research foundation.

## CONFLICT OF INTEREST STATEMENT

BS reports financial support for travel grants from Gilead Sciences and ViiV healthcare, and for advisory boards from Gilead Sciences and MSD, paid to his institution. DH reports consultancies: AstraZeneca, Gilead, UCB, ViiV Healthcare; travel grants: Gilead; institutional funding: AstraZeneca, Gilead, GSK, A. Menarini Pharma, MSD, Pfizer, ViiV Healthcare. All other authors had no conflicts of interest.

## Supporting information


**File S1.** Eligibility criteria.
**File S2.** Full questionnaire in English version (attached).
**File S3.** GRIPP2 short form for reporting on Patient and Public Involvement.
**File S4.** Sociodemographic and virologic characteristics of study sample compared to all eligible^$^ participants.
**File S5.** Factors associated with reported overall burden of treatment.
**File S6.** Factors associated with having heard about LA‐ART previously.
**File S7.** Factors associated with willing to stay on oral ART and not being interested to know more about iLA‐ART.
**File S8.** Factors associated with overall satisfaction about current oral ART.
**File S9.** Factors associated with knowing ≥ 50% of the correct answers to the ‘knowledge’ questions on LA‐ART*.

**File 2.** Full Questionnaire.

## Data Availability

The individual‐level datasets generated or analysed during the current study do not fulfil the requirements for open data access: (1) The SHCS informed consent states that sharing data outside the SHCS network is only permitted for specific studies on HIV infection and its complications, and to researchers who have signed an agreement detailing the use of the data and biological samples; and (2) The data is too dense and comprehensive to preserve patient privacy in people living with HIV. According to Swiss law, data cannot be shared if individuals have not agreed or data are too sensitive to share. Investigators with a request for selected data should send a proposal to the respective SHCS address (www.shcs.ch/contact). The provision of data will be considered by the Scientific Board of the SHCS and the study team, and is subject to Swiss legal and ethical regulations and is outlined in a material and data transfer agreement.
