# Supplementary material for: Do people with HIV really have the choice between oral and injectable ART? Evidence from a multicentric survey in the Swiss HIV cohort study
Source: HIV Med. 2026 Apr 8;27(7):1156–67. doi: 10.1111/hiv.70239 (PMC13340992; doi:10.1111/hiv.70239)
Supplement: Supplementary file 1 — File S1. Eligibility criteria. File S2. Full questionnaire in English version (attached). File S3. GRIPP2 short form for reporting on Patient and Public Involvement. File S4. Sociodemographic and virologic characteristics of study sample compared to all eligible$ participants. File S5. Factors associated with reported overall burden of treatment. File S6. Factors associated with having heard about LA‐ART previously. File S7. Factors associated with willing to stay on oral ART and not being interested to know more about iLA‐ART. File S8. Factors associated with overall satisfaction about current oral ART. File S9. Factors associated with knowing ≥ 50% of the correct answers to the ‘knowledge’ questions on LA‐ART*. [file HIV-27-1156-s002.docx]

**Supporting Information Files**

**Supporting Information File 1**: Eligibility criteria

1. Inclusion criteria

– Age ≥18 y.o.

– At enrolment on oral ART

1. Exclusion criteria

- HBV coinfection
- HIV Subtype A6/A1
- Severe liver disease
- Known resistance to INSTI (T66I, E92Q, G118R, F121Y, G140R, N155H, R263K) or NNRTIs (L100I, K101E/P, E138A/G/K/Q/R, F227C, M230L/I), in analogy to SOLAR study
- Virological failure in the last 6 months
- Patient on anticoagulation
- Patient on strong UGT1A1 or CYP3A inductors such as Rifampicin, Rifapentin, Rifabutin, Phenytoin, Phenobarbital, Carbamazepin, Oxcarbazepin, Dexamethason and Saint-John’s-wort

**Supporting Information File 2.** Full questionnaire in English version (attached)

^
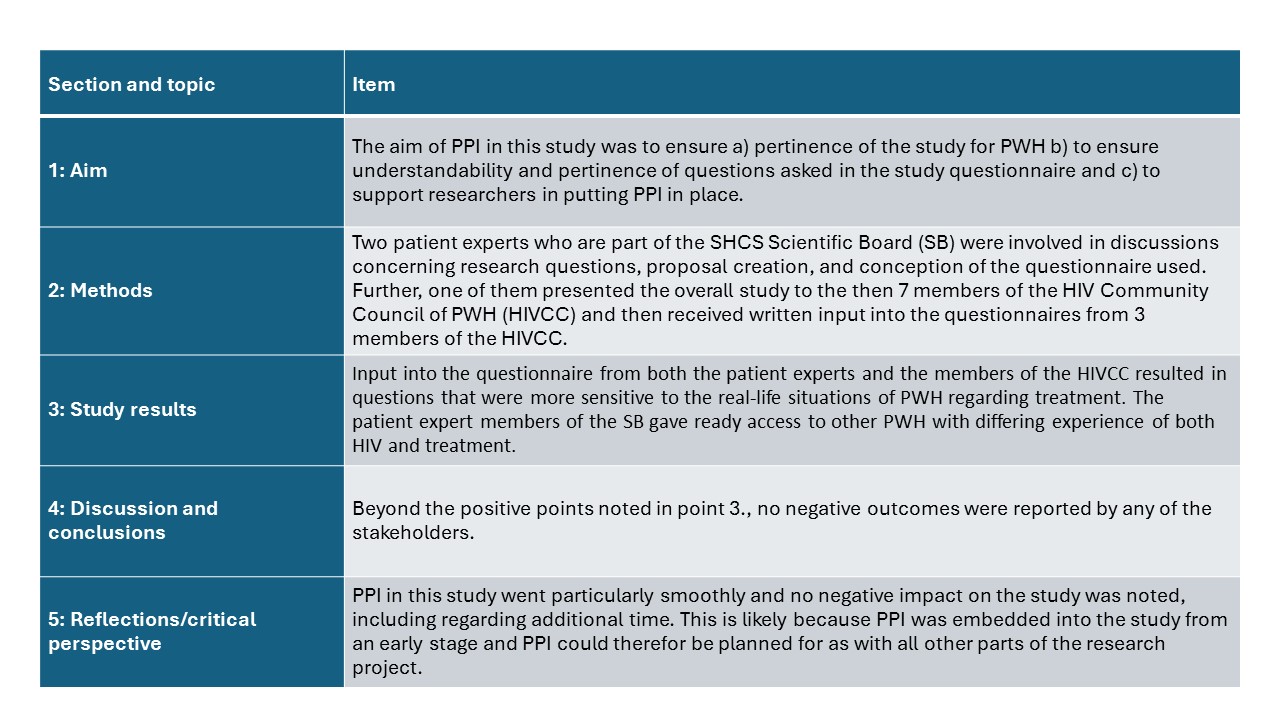
^**Supporting Information File 3.** GRIPP2 short form for reporting on Patient and Public Involvement

| **Characteristic** | **Eligible included patients, n = 200 (%)** | **Eligible not included patients^†^, n= 1’810 (%)** | **p value** |
| --- | --- | --- | --- |
| **Women** | 58 (29) | 599 (33) | 0.24 |
| **Age category**  18-39 years  40-59 years  ≥60 years | 28 (14)  122 (61)  50 (25) | 263 (14)  1’027 (57)  520 (29) | 0.47 |
| **Transmission group**  Heterosexual  PWID  MSM  Other  Missing | 88 (44)  12 (6)  90 (45)  8 (4)  2 (1) | 686 (38)  179 (10)  826 (46)  119 (7)  - | 0.06 |
| **Ethnicity**  White  Other  Missing | 153 (77)  45 (23)  2 (1) | 1'247 (69)  561 (31)  - | 0.02 |
| **Highest level of education**  Mandatory school or less  Apprenticeship or any degree  Missing | 42 (22)  153 (78)  5 (2) | 506 (28)  1’272 (70)  - | 0.04 |
| **Current oART**  Single pill regime  At least two pills | 172 (86)  27 (14) | 1’263 (70)  547 (30) | <0.001 |
| **Center**  1  2  3 | 107 (53)  67 (34)  26 (13) | 748 (41)  977 (54)  85 (5) | <0.001 |
| **Last HIV Viral load**  <50 cp/mL  >50 cp/mL | 195 (98)  5 (2) | 1’700 (94)  107 (6) | 0.04 |
| **CD4 Nadir**  <200 cells/µL  ≥200 cells/µL | 85 (43)  115 (57) | 826 (46)  984 (54) | 0.40 |
| **ART duration, median (IQR)**  <10 years  ≥10 years | 14.3 (8.6 – 20.0)  61 (31)  139 (69 | 16.3 (9.4 – 24.5)  499 (28)  1’311 (72) | 0.38 |

**Supporting Information File 4. Sociodemographic and virologic characteristics of study sample compared to all eligible^$^ participants.**

**PWID**: People who inject drugs; **MSM**: Men who have sex with men, **ART**: antiretroviral therapy

**^†^**Eligible patients for iLA-ART: SHCS participants in care in one of the participating centres and who had no evidence of virological failure in the last 6 months and no HBV co-infection.

**Supporting Information File 5.** **Factors associated with reported overall burden of treatment.**

|  | Univariate analysis | | Multivariable analysis | |
| --- | --- | --- | --- | --- |
|  | Coeff. (95% CI) | *P* | Coeff. (95% CI) | *P* |
| Gender  Men  Women | Reference  0.07 (-0.59-0.73) | *0.83* | Reference  -0.36 (-1.16-0.44) | *0.37* |
| Age Category  18-39 years  40-59 years  ≥60 years | Reference  0.22 (-0.67-1.10)  0.06 (-0.93-1.05) | *0.83* | Reference  -0.12 (-0.99-0.76)  -0.12 (-1.11-0.86) | *0.96* |
| Transmission group  MSM  Heterosexual  Other | Reference  0.50 (-0.11-1.12)  1.14 (0.18-2.09) | *0.04* | Reference  0.44 (-0.34-1.23)  0.97 (-0.11-2.05) | *0.19* |
| Ethnicity  White  Other | Reference  0.31 (-0.42-1.05) | *0.40* |  |  |
| Highest level of education  Mandatory school or less  Apprenticeship or any degree | Reference  -1.03 (-1.75- -0.31) | *0.01* | Reference  -1.03 (-1.78- -0.27) | *0.01* |
| Current ART  1 pill regimen  ≥2 pills regimen | Reference  -0.81 (-1.64-0.03) | *0.06* | Reference  -0.70 (-1.54-0.14) | *0.10* |
| Center  1  2  3 | Reference  -0.77 (-1.39- -0.15) 0.80 (-0.27-1.88) | *0.01* | Reference  -0.94 (-1.58- -0.31)  0.82 (-0.32-1.95) | *<0.01* |
| CD4 Nadir  <200 cells/µL  ≥200 cells/µL | Reference  -0.15 (-0.74-0.44) | *0.61* |  |  |
| ART duration  <10 years  ≥10 years | Reference  0.45 (-0.18-1.09) | 0.16 |  |  |

**PWID**: People who inject drugs; **MSM**: Men who have sex with men, **ART**: antiretroviral therapy

**Supporting Information File 6.** **Factors associated with having heard about LA-ART previously.**

|  | Univariate analysis | | Multivariable analysis | |
| --- | --- | --- | --- | --- |
|  | OR (95% CI) | *P* | OR (95% CI) | *P* |
| Gender  Men  Women | Reference  0.63 (0.33-1.18) | *0.08* | Reference  0.35 (0.14-0.85) | *0.02* |
| Age Category  18-39 years  40-59 years  ≥60 years | Reference  0.63 (0.24-1.60)  0.28 (0.10-0.80) | *0.02* | Reference  0.73 (0.24-2.21)  0.27 (0.08-0.94) | *0.05* |
| Transmission group  MSM  Heterosexual  Other | Reference  0.54 (0.29-1.00)  0.67 (0.26-1.76) | *0.14* |  |  |
| Ethnicity  White  Other | Reference  1.69 (0.82-3.50) | *0.15* |  |  |
| Highest level of education  Mandatory school or less  Apprenticeship or any degree | Reference  0.52 (0.25- 1.09) | *0.08* | Reference  0.60 (0.23-1.56) | *0.29* |
| Current ART  1 pill regimen  ≥2 pills regimen | Reference  0.66 (0.29-1.49) | *0.32* |  |  |
| Center  1  2  3 | Reference  47.24 (10.96-203.58) 1.48 (0.61-3.60) | *<0.001* | Reference  52.02 (11.48-235.75)  1.28 (0.48-3.43) | *<0.001* |
| CD4 Nadir  <200 cells/µL  ≥200 cells/µL | Reference  1.56 (0.87-2.80) | *0.13* |  |  |
| ART duration  <10 years  ≥10 years | Reference  0.60 (0.32-1.15) | 0.12 |  |  |

**PWID**: People who inject drugs; **MSM**: Men who have sex with men, **ART**: antiretroviral therapy

|  | Univariate analysis | | Multivariable analysis | |
| --- | --- | --- | --- | --- |
|  | OR (95% CI) | *P* | OR (95% CI) | *P* |
| Gender  Men  Women | Reference  1.17 (0.57-2.38) | *0.67* | Reference  1.23 (0.59-2.56) | *0.58* |
| Age Category  18-39 years  40-59 years  ≥60 years | Reference  1.21 (0.48-3.08)  2.78 (0.97-7.94) | *0.06* | Reference  1.17 (0.46-3.00)  2.75 (0.96-7.87) | *0.06* |
| Transmission group  MSM  Heterosexual  Other | Reference  1.57 (0.80-3.09)  0.59 (0.19-1.81) | *0.15* |  |  |
| Ethnicity  White  Other | Reference  0.67 (0.30-1.50) | *0.33* |  |  |
| Highest level of education  Mandatory school or less  Apprenticeship or any degree | Reference  0.83 (0.37- 1.86) | *0.66* |  |  |
| Current ART  1 pill regimen  ≥2 pills regimen | Reference  1.02 (0.41-2.52) | *0.97* |  |  |
| Center  1  2  3 | Reference  1.77 (0.89-3.52)  1.63 (0.58-4.58) | *0.23* |  |  |
| CD4 Nadir  <200 cells/µL  ≥200 cells/µL | Reference  0.71 (0.37-1.35) | *0.30* |  |  |
| ART duration  <10 years  ≥10 years | Reference  1.21 (0.61-2.42) | 0.58 |  |  |

**Supporting Information File 7.** **Factors associated with** **willing to stay on oral ART and not being interested to know more about iLA-ART.**

**PWID**: People who inject drugs; **MSM**: Men who have sex with men, **ART**: antiretroviral therapy

**Supporting Information File 8.** **Factors associated with overall satisfaction about current oral ART.**

|  | Univariate analysis | | Multivariable analysis | |
| --- | --- | --- | --- | --- |
|  | Coeff. (95% CI) | *P* | Coeff. (95% CI) | *P* |
| Gender  Men  Women | Reference  -0.04 (-0.46-0.37) | *0.84* | Reference  -0.14 (-0.55-0.27) | *0.50* |
| Age Category  18-39 years  40-59 years  ≥60 years | Reference  0.92 (0.38-1.46)  1.03 (0.42-1.63) | *0.002* | Reference  0.88 (0.32-1.44)  0.91 (0.27-1.55) | *0.007* |
| Transmission group  MSM  Heterosexual  Other | Reference  -0.01 (-0.41-0.38)  -0.49 (-1.12-0.14) | *0.29* |  |  |
| Ethnicity  White  Other | Reference  -0.19 (-0.65-0.26) | *0.40* |  |  |
| Highest level of education  Mandatory school or less  Apprenticeship or any degree | Reference  0.27 (-0.19- 0.74) | *0.25* |  |  |
| Current ART  1 pill regimen  ≥2 pills regimen | Reference  0.21 (-0.33-0.75) | *0.44* |  |  |
| Center  1  2  3 | Reference  -0.15 (-0.55-0.25) -0.55 (-1.20-0.10) | *0.24* |  |  |
| CD4 Nadir  <200 cells/µL  ≥200 cells/µL | Reference  -0.39 (-0.77-0.02) | *0.04* | Reference  -0.23 (-0.62-0.15) | *0.23* |
| ART duration  <10 years  ≥10 years | Reference  0.10 (-0.31-0.51) | 0.63 |  |  |

**PWID**: People who inject drugs; **MSM**: Men who have sex with men, **ART**: antiretroviral therapy

**Supporting Information File 9. Factors associated with knowing ≥50% of the correct answers to the “knowledge” questions on LA-ART*.**

|  | Univariate analysis | | Multivariate analysis | |
| --- | --- | --- | --- | --- |
|  | OR (95% CI) | *P* | OR (95% CI) | *P* |
| Gender  Men  Women | Reference  1.14 (0.47-2.72) | *0.29* | Reference  1.57 (0.51-4.78) | *0.43* |
| Age Category (years)  18-39  40-59  ≥60 | Reference  1.63 (0.61-4.38)  1.78 (0.52-6.03) | *0.57* | Reference  1.48 (0.49-4.41)  1.81 (0.43-7.65) | *0.69* |
| Transmission group  MSM  Heterosexual  Other | Reference  0.61 (0.27-1.37)  0.48 (0.06-2.00) | *0.35* |  |  |
| Ethnicity  White  Other | Reference  0.48 (0.20-1.13) | *0.09* | Reference  0.36 (0.12-1.07) | *0.07* |
| Highest level of education  Mandatory school or less  Apprenticeship or any degree | Reference  1.03 (0.43- 2.46) | *0.95* |  |  |
| Current ART  1 pill regimen  ≥2 pills regimen | Reference  1.26 (0.38-4.26) | *0.70* |  |  |
| Center  1  2  3 | Reference  5.68 (2.32-13.91)  0.76 (0.17-3.35) | *<0.001* | Reference  5.74 (2.25-14.69)  0.79 (0.16-3.81) | *<0.001* |
| CD4 Nadir (cells/µL)  <200  ≥200 | Reference  1.32 (0.61-2.86) | *0.48* |  |  |
| ART duration (years)  <10  ≥10 | Reference  0.89 (0.41-1.93) | 0.77 |  |  |

*Analysis performed only among participants who mentioned having heard about LA-ART previously.

**PWID**: People who inject drugs; **MSM**: Men who have sex with men, **ART**: antiretroviral therapy

**OR**: Odds Ratio
