# Supplementary material for: Do people with HIV really have the choice between oral and injectable ART? Evidence from a multicentric survey in the Swiss HIV cohort study
Source: HIV Med. 2026 Apr 8;27(7):1156–67. doi: 10.1111/hiv.70239 (PMC13340992; doi:10.1111/hiv.70239)
Supplement: Supplementary file 2 — File 2. Full Questionnaire. [file HIV-27-1156-s001.docx]

**Supporting Information File 2.**

**Full Questionnaire**

SHCS number: _____________

Current treatment (ART) : _____________

1. On a scale of 0 to 10, what is your level of satisfaction with your current treatment?

(1: not satisfied at all, 10: fully satisfied)? _____________

1. Which aspect of your HIV treatment do you like the least?

____________________________________________________________________

1. Are the people living in the same household aware that you are on medications against HIV ?

*Yes Some of them, but not all No I live alone*

**Which of the following criteria regarding your HIV medication are important to you** :

Choose a number between 0 and 10 **(0 = Totally disagree and 10 = Fully agree)**

1. Number of tablets (as few as possible)

*0 - 1 - 2 - 3 - 4 - 5 - 6 - 7 -8 - 9 - 10 No opinion*

1. Can be taken once a day

*0 - 1 - 2 - 3 - 4 - 5 - 6 - 7 -8 - 9 - 10 No opinion*

1. To have as few side effects as possible

*0 - 1 - 2 - 3 - 4 - 5 - 6 - 7 -8 - 9 - 10 No opinion*

1. Less risk for interactions with other medications

*0 - 1 - 2 - 3 - 4 - 5 - 6 - 7 -8 - 9 - 10 No opinion*

1. General effectiveness of the drug

*0 - 1 - 2 - 3 - 4 - 5 - 6 - 7 -8 - 9 - 10 No opinion*

1. The treatment remains effective despite forgetting taking it once or few time

*0 - 1 - 2 - 3 - 4 - 5 - 6 - 7 -8 - 9 - 10 No opinion*

1. Treatment price

*0 - 1 - 2 - 3 - 4 - 5 - 6 - 7 -8 - 9 - 10 No opinion*

1. Assures discretion (flexibility of intake time, package size)

*0 - 1 - 2 - 3 - 4 - 5 - 6 - 7 -8 - 9 - 10 No opinion*

1. Tablet size – as small as possible

*0 - 1 - 2 - 3 - 4 - 5 - 6 - 7 -8 - 9 - 10 No opinion*

1. Treatment compatibility with pregnancy and breast feeding

*0 - 1 - 2 - 3 - 4 - 5 - 6 - 7 -8 - 9 - 10 No opinion*

**Knowledge injectables:**

1. Have you ever heard about injectable therapy against HIV?

*Yes No 🡪If no, skip this section and move to “preferences on injectables”*

**If the patient doesn’t know the answer and tries to guess, choose « I don’t know ».**

1. Treatment with injectable cabotegravir/rilpivirin is as effective as modern oral HIV medication?

*Yes No, it is more effective No, it is less effective I don’t know*

1. What are the possible intervals between injections (with injectables currently available in Switzerland)?

*2 (or 1) months 3 m. 6 m. 1 year I don’t know*

1. How should a treatement with cabotegravir/rilpivirin be administered?

*Subcutaneously Intramuscularly Intravenously I don’ know*

1. In which part of the body are the injections generally administered?

*The arm The thigh The belly The buttock*

1. In which setting do the cabotegravir/rilpivirin injections need to be administered ?

*At home by myself*

*At home by nurses*

*At my GP (general practionner) practice*

*At my HIV care center*

*I don’t know*

1. What is the most frequent side effect related to treatment with injectable cabotegravir/rilpivirin ?

*Local site reactions*

*Nausea/vomiting*

*Headache*

*Skin rash*

*I don’t know*

1. How often is this side effect observed ?

*Rarely (<10%) Regularly (10-30%) Frequently (>50%) I don’t know*

1. The treatment price for injectable cabotegravir/rilpivirin is in the same range as modern oral HIV medication ?

*Yes No, much higher No, much lower I don’t know*

1. Do the injections have to be performed at fixed intervals?

*Yes No, the intervals are flexible I don’t know*

1. If I forget my appointment for the injections and/or if I can’t get my injections within 7 days after the planned injection, what will be the consequence for me?

*None, I will receive the injection whenever I can.*

*I can’t resume my therapy and need to stop injectable treatment.*

*I need to take an oral therapy containing the same substances as the injected ones until I can go to get my injections.*

*I don’t know*

**Preferences on injectables:**

If in your situation an injectable therapy with cabotegravir/rilpivirin administered every 2 months would be possible instead of the tablet(s) you daily take, how do you evaluate the following points :

I’d be interested in such a therapy and I’d like to know more.

*Yes I don’t know No, I prefer to take (a) tablet(s)*

**Section A -** Choose a number between 0 and 10 **(0 = Totally disagree and 10 = Fully agree)**

1. For me, not having to take (a) tablet(s) daily would mean more freedom.

*0 - 1 - 2 - 3 - 4 - 5 - 6 - 7 -8 - 9 - 10 No opinion*

1. On the contrary, having to get injections on fixed dates would mean less freedom to me.

*0 - 1 - 2 - 3 - 4 - 5 - 6 - 7 -8 - 9 - 10 No opinion*

1. Because no one would see me take tablets anymore, to be on injectable treatment would improve my privacy.

*0 - 1 - 2 - 3 - 4 - 5 - 6 - 7 -8 - 9 - 10 No opinion*

1. On the contrary, having to go every 2 months – and thus more often than what I currently do – to the hospital would have a **negative effect on my privacy and/or professional life.**

*0 - 1 - 2 - 3 - 4 - 5 - 6 - 7 -8 - 9 - 10 No opinion*

1. Coming to the hospital every 2 months (to get the injections) instead of every 3 or 6 months is **not desirable** for me.

*0 - 1 - 2 - 3 - 4 - 5 - 6 - 7 -8 - 9 - 10 No opinion*

1. Having to remember to take medication daily reminds me that I have HIV and I experience this negatively.

*0 - 1 - 2 - 3 - 4 - 5 - 6 - 7 -8 - 9 - 10 No opinion*

1. I would be worried about getting intramuscular injections.

*0 - 1 - 2 - 3 - 4 - 5 - 6 - 7 -8 - 9 - 10 No opinion*

1. I would be worried about side effects at injections sites.

*0 - 1 - 2 - 3 - 4 - 5 - 6 - 7 -8 - 9 - 10 No opinion*

1. The recommended injection site (the buttock) would be embarrassing for me.

*0 - 1 - 2 - 3 - 4 - 5 - 6 - 7 -8 - 9 - 10 No opinion*

1. The fact that the injectable treatment cannot be injected by myself at home is a real barrier to this kind of treatment.

*0 - 1 - 2 - 3 - 4 - 5 - 6 - 7 -8 - 9 - 10 No opinion*

**Section B -** Choose a number between 0 and 10 **(0 = Totally disagree and 10 = Fully agree)**

A few reasons which mean I would prefer to continue on oral therapy:

1. Taking a tablet/tablets every day against HIV is part of my daily routine.

*0 - 1 - 2 - 3 - 4 - 5 - 6 - 7 -8 - 9 - 10 No opinion*

1. Being able to decide when is the best time to take my tablet/s (flexibility) is of great importance to me.

*0 - 1 - 2 - 3 - 4 - 5 - 6 - 7 -8 - 9 - 10 No opinion*

1. I do not want to increase the yearly number of hospital visits (for the injections).

*0 - 1 - 2 - 3 - 4 - 5 - 6 - 7 -8 - 9 - 10 No opinion*

1. Needing to get the injection in a hospital or practice setting would make me feel that I have a ‘serious illness’.

*0 - 1 - 2 - 3 - 4 - 5 - 6 - 7 -8 - 9 - 10 No opinion*

1. I already take several other (non-HIV) tablets, so taking my HIV treatment does not really make a difference.

*0 - 1 - 2 - 3 - 4 - 5 - 6 - 7 -8 - 9 - 10 No opinion*

1. I am worried about switching to another kind of treatment, as my HIV has always been well controlled with tablets (loss of control).

*0 - 1 - 2 - 3 - 4 - 5 - 6 - 7 -8 - 9 - 10 No opinion*

1. The frequency of injections is an important barrier to me. If they could be administered every 6 months instead of every 2 months, I’d be interested in such a treatment.

*0 - 1 - 2 - 3 - 4 - 5 - 6 - 7 -8 - 9 - 10 No opinion*

**Burden of Treatment**

Choose a number between 0 and 10

**(0 = Not constraining at all and 10 = Very constraining)**

1. To what extent is it constraining to have to remind you taking your HIV tablet(s) every day/to remind you presenting yourself for your injections visits?

*0 - 1 - 2 - 3 - 4 - 5 - 6 - 7 -8 - 9 - 10*

1. The side effects related to my HIV treatment (Tablets or injections) are:

*0 - 1 - 2 - 3 - 4 - 5 - 6 - 7 -8 - 9 - 10*

1. In case of travelling, prolonged leave or missed appointment, organizing my HIV treatment is:

*0 - 1 - 2 - 3 - 4 - 5 - 6 - 7 -8 - 9 – 10*

1. How do you evaluate the **time** spent on medical visits (**number of visits** **and time spent** during the visits)?

*0 - 1 - 2 - 3 - 4 - 5 - 6 - 7 -8 - 9 - 10*

1. To what extent is it constraining to **organize your diary** to be able to attend your medical visits?

*0 - 1 - 2 - 3 - 4 - 5 - 6 - 7 -8 - 9 - 10*

1. The intake/injection of my medication has an influence on my life with other people (taking tablets in public, embarrassment, absenteeism related to my medical visits)?

*0 - 1 - 2 - 3 - 4 - 5 - 6 - 7 -8 - 9 - 10*
